# Reproducibility of surface-based deep inspiration breath-hold technique for lung stereotactic body radiotherapy on a closed-bore gantry linac

**DOI:** 10.1016/j.phro.2023.100448

**Published:** 2023-05-13

**Authors:** Daniel Nguyen, Rebeca Reinoso, Jad Farah, Sena Yossi, Fabrice Lorchel, Victor Passerat, Estelle Louet, Isabelle Pouchard, Mustapha Khodri, Nicolas Barbet

**Affiliations:** a ORLAM’s Group, Department of Radiation Oncology, Mâcon, Villeurbanne, Lyon, France; b Vision RT Ltd., Dove House, Arcadia Avenue, London N3 2JU, United Kingdom

**Keywords:** Deep-inspiration breath-hold, Lung stereotactic body radiotherapy, Ring-mounted SGRT system, Intra-fraction control

## Abstract

**Background and purpose:**

Tumor motion and delivery efficiency are two main challenges of lung stereotactic body radiotherapy (SBRT). The present work implemented the deep inspiration breath hold technique (DIBH) with surface guided radiation therapy (SGRT) on closed-bore linacs and investigated the correlation between SGRT data and internal target position.

**Materials and methods:**

Thirteen lung SBRT patients treated in DIBH using a closed-bore gantry linac and a ring-mounted SGRT system were retrospectively analysed. Visual coaching was used to achieve DIBH with a ± 1 mm threshold window in the anterior-posterior direction. Three kV-CBCTs were added to the treatment workflow and examined offline to verify intra-fraction tumor position. Surface-based DIBH was analysed using SGRT treatment reports and an in-house python script. Data from 73 treatment sessions and 175 kV-CBCTs were studied. Correlations between target and surface positions were studied with Linear Mixed Models.

**Results:**

Median intra-fraction tumor motion was 0.8 mm (range: 0.7–1.3 mm) in the anterior-posterior direction, 1.2 mm (range: 1–1.7 mm) in the superior-inferior direction, and 1 mm (range: 0.7–1.1 mm) in the left–right direction, with rotations of <1° (range: 0.6°–1.1°) degree in all three directions. Planned target volumes and healthy lung volumes receiving 12.5 Gy and 13.5 Gy were reduced on average by 67% and 54%, respectively.

**Conclusions:**

Lung SBRT in DIBH with the ring-mounted SGRT system proved reproducible. The surface monitoring provided by SGRT was found to be a reliable surrogate for internal target motion. Moreover, the implementation of DIBH technique helped reduce target volumes and lung doses.

## Highlights

• Lung stereotactic body radiotherapy was performed in deep-inspiration breath-hold on a closed-bore linac, with improved treatment times and patient comfort.
• Computed tomography scanning confirmed reproducible breath-hold, with tumor within 1.5 mm and 1.5°.
• Planning target volumes were reduced by 67%, with consequently large reduction in healthy lung exposure.

## 1 Introduction

Lung cancer is the second most common and the leading cause of cancer death worldwide in 2020 [1] with 80–90% of lung cancer patients having non-small cell lung cancer (NSCLC) [2], which is conventionally managed by surgical resection [3]. Meanwhile, stereotactic body radiotherapy (SBRT) represents an invaluable alternative for the medically inoperable early-stage lung cancer population [3]. However, to reduce the effects of respiratory tumor motion during lung SBRT, an internal target volume (ITV) generated from 4D computed tomography scan (4D-CT) is commonly applied. This approach can result in an overestimated planning target volume (PTV) and does not consider respiratory variations, ITV definition instabilities, or interplay effects during treatment delivery [4], [5], [6].

The deep inspiration breath-hold (DIBH) technique represents another alternative approach to limit tumor motion [7], [8] minimise PTV and significantly decrease doses to organs at risk (OAR) [7], [8], [9]. In addition, the use of intrafractional monitoring techniques allows further reduction of the planning target volume such as electromagnetic transponders [10], spirometer-based systems [11], opto-electronic systems [9], or surface-guided radiotherapy (SGRT) [12], [13] managing the residual target motion during DIBH more effectively. SGRT devices are increasingly used to perform voluntary DIBH with visual guidance to the patients [11], [13]. They have been primarily applied to left-sided breast cancer [12], [14], [15], [16] and more recently for lung and liver SBRT [9], [11].

However, the implementation of SGRT-guided DIBH for lung SBRT treatments can represent a significant challenge given the required sub-mm accuracy in tumor position. Additionally, while lung cancer patients suffer from respiratory failures/distress/insufficiencies, treating these specific pathologies in DIBH is even more challenging when multiple BHs are required for cone-beam CT (CBCT) acquisition (generally 2–3) on C-arm linacs [11], [16]. Closed-bore gantry linac offering fast kV-CBCT imaging and high dose delivery rates would allow such an assessment when combined with the proper SGRT system. Indeed, some authors have attempted to apply DIBH for left breast cancer using two ceiling-mounted SGRT cameras positioned at the front or the back of a closed-bore gantry linac [17], [18], [19] showing limited surface coverage which was deemed insufficient for SBRT treatments. Meanwhile, a dedicated ring-mounted SGRT system has been recently introduced and proven to allow six degrees-of-freedom (DoF) intra-fraction motions monitoring inside the linac bore/tunnel with full surface coverage [12], [13].

The present work combined the benefits of the closed-bore gantry linac and the dedicated ring-mounted SGRT system, and implemented lung SBRT treatments with the voluntary DIBH technique as the new routine practice for such cancer treatments on closed-bore gantry linacs. The study hence aimed at evaluating the reproducibility of internal tumor position during successive SGRT-guided DIBH manoeuvres and the benefit for patients in terms of dose/volume metrics.

## 2 Materials and Methods

### 2.1 Patient population and coaching session

Thirteen consecutive lung SBRT patients treated between March 2021 and October 2022 on Halcyon™ (6 MV FFF beams) with the SGRT-based abdominal DIBH (aDIBH) technique were included in this study. During the medical consultation, the radiation oncologist explained to the patient the aDIBH procedure, which involves relaxing the shoulders and pushing out the belly while keeping the diaphragm in a fixed position [20]. Patients able to hold their breath for at least 15 s, i.e. the duration of the kV-CBCT acquisition on Halcyon™ (Varian, Palo Alto, USA), were deemed eligible. Patients’ informed consent was obtained, and institutional review board approval was waived.

For all patients, a 4D-CT scan was first performed using an Aquilion LB™ CT scan (Canon Medical Systems, Ōtawara, JP) and a 1 mm slice thickness. Based on these 4DCT images, the DIBH technique was considered whenever tumor motion exceeded 5 mm and the patient was able to hold their breath for 15 s (cf. Supplementary Material A). A DIBH coaching session was then performed in the CT room using a single-camera pod and AlignRT™ software v.5.2 (Vision RT Ltd., London, UK). Audio and visual patient coaching were provided by the Real-Time Coach™ (RTC) [12] with a set window of +/-1 mm for the vertical value and +/-2 mm/° for the other directions, these thresholds were also used during treatment. Multiple breath holds (BHs) were repeated and the amplitude of the 6 Real-Time Delta (RTD) values and duration of each BH were measured to confirm DIBH reproducibility, stability and the patient’s eligibility for SBRT treatment with such a technique.

Table 1 documents some of the key tumor and patient characteristics, dose information as well as treatment times. The mean thoracic elevation was 12 ± 3 mm with a range of comfortable DIBH of 15–20 s. Patients performed 12 to 15 DIBH per treatment session.

**Table 1:** SBRT DIBH patient information. “N° of MU” shows the MU number for the overall treatment course. The “treatment time (min)” presents the median and the interquartile range (Q1 -Q3) of the treatment time.

**Patient No**	**Tumor Localisation**	**Sex**	**Age**	**Weight (kg)**	**PTV FB (cm3)**	**PTV DIBH (cm3)**	**Dose (Gy)**	**N° fraction**	**N° arcs**	**N° of MU/fraction**	**Anterior SSD (cm)**	**Treatment time (min)**
**1**	Upper lobe	Female	81	71	41.7	22.3	55	5	6	9688	86.3	26.4 (17.9–30.1)
**2**	Inferior lobe	Male	75	92	55.9	18.7	52.5	7	6	1712	80.1	15.8 (14.4–23.8)
**3**	Inferior lobe	Male	77	71	25.4	21.7	55	5	6	2728	91.7	22.4 (22.3–27.5)
**4**	Inferior lobe	Male	86	74	26.1	13.9	55	5	4	2592	81.1	21.9 (21.9–22.8)
**5**	Inferior lobe	Male	86	74	–	20.6	55	5	6	3067	80.8	16.7 (15.1–19.2)
**6**	Inferior lobe	Male	54	55	8.6	5.7	50	5	4	1774	86.3	23.9 (19.9–27.4)
**7**	Inferior lobe	Male	54	55	–	78.9	50	5	4	2227	88.3	18.9 (14.5–21.7)
**8**	Upper lobe	Male	86	76	–	21.7	60	8	6	3230	89	20.2 (20–21.6)
**9**	Middle lobe	Female	79	107	21.9	17.5	60	8	6	3617	86.9	28.8 (25.8–31.4)
**10**	Inferior lobe	Male	65	60	9.6	7.6	50	5	4	2319	88.3	14.4 (12.5–15.1)
**11**	Inferior lobe	Male	79	72	51.4	45.7	55	5	6	2375	85.6	24.2 (18.8–25.1)
**12**	Middle lobe	Female	44	102	90.4	68.3	55	5	6	3519	81.9	20.8 (19.6–24.6)
**13**	Upper lobe	Male	86	74	77.6	47	55	5	4	2149	80.9	20.7 (18.4–20.9)

Abbreviations: PTV: planning target volume, FB: free breathing, DIBH: deep inspiration breath-hold.

### 2.2 CT acquisition and treatment planning

The initial treatment plan was calculated using the CT average from the 4D-CT. Only three patients did not receive a 4D-CT following a medical decision. If the plan met the Critical Dose-Volume Limits of RTOG recommendations and criteria [21], [22], [23] the treatment delivery was done in free breathing (FB). Otherwise, two days after the coaching session, a FB and a DIBH CT acquisition were acquired with AlignRT™ and RTC™ guidance (Supplementary Material A). The DIBH CT is used to perform the dosimetry following the same recommendations [21], [22], [23]. FB CT is only used for patient setup with AlignRT InBore™.

The contouring of organs at risk (OARs) was performed by the radiation oncologist and applied a 5 mm isotropic expansion of the CTV to obtain the PTV. Treatment planning was performed with the Raystation-11B treatment planning system (TPS) and collapsed cone convolution (CCC) algorithm (Raysearch, Stockholm, SWE) with a 1 mm grid resolution. Doses to OAR were evaluated following RTOG 0813 [21], UK 2022 SABR [22] and RECORAD [23] recommendations. The ratio between the lung volume receiving 12.5 or 13.5 Gy and the total normal lung volume was calculated from the average 4D-CT and the DIBH CT. Target coverage criteria were in accordance with RTOG 0813 [21] as follows, at least 95% of the PTV received the prescribed dose with a minimum of 90% of the dose delivered to at least 99% of the PTV. The prescribed dose limit was set at a maximum of 120%.

### 2.3 Setup verification and treatment delivery

Reference patient position in FB and DIBH were recovered by importing the DICOM-RT plan and structures into AlignRT® InBore v6.3 (Vision RT Ltd., London, UK). Ceiling-mounted cameras were first used for patient setup with two ROIs for each patient (cf. Supplementary Material B). The FB setup ROI covered the entire chest while the DIBH treatment ROI contained the ipsilateral part of the thorax overlapping the isocenter. The larger ROI used in FB was designed for initial setup, whereas the smaller ROI in DIBH is used for setup refinement and takes in consideration the narrower FOV of the AlignRT Inbore cameras [10]. Next, patient’s ability to perform DIBH was confirmed with ceiling mounted cameras at setup position then using the ring-mounted cameras at treatment position. During imaging and treatment delivery, the patient was instructed to perform BHs using the intercom.

Four kV-CBCTs acquisitions per treatment fraction were considered to verify tumor position throughout the treatment. The first mandatory kV-CBCT was used to verify tumor position and overall patient alignment based on DIBH planning CT. Following image matching (bony anatomy and soft tissue) and analysis, couch shifts were applied with the patient in DIBH and a new reference surface was captured with AlignRT InBore™. Subsequently, three control kV-CBCTs were taken prior to the first treatment delivery, halfway through the treatment and before the last VMAT arc without applying any couch shifts following such additional X-ray images.

### 2.4 Data analysis of tumor position

Control kV-CBCTs were retrospectively analysed offline to verify target positions individually. The shifts were calculated by comparing the control kV-CBCT and the planning CT in DIBH using Eclipse (Varian Medical Systems, Palo Alto, USA) to obtain the values in the six DoF. The amplitude of target movement during treatment was calculated as the difference between the maximum and the minimum value of the computed shifts across the three control kV-CBCTs.

### 2.5 Data analysis of surface position

**Fig. 1:**
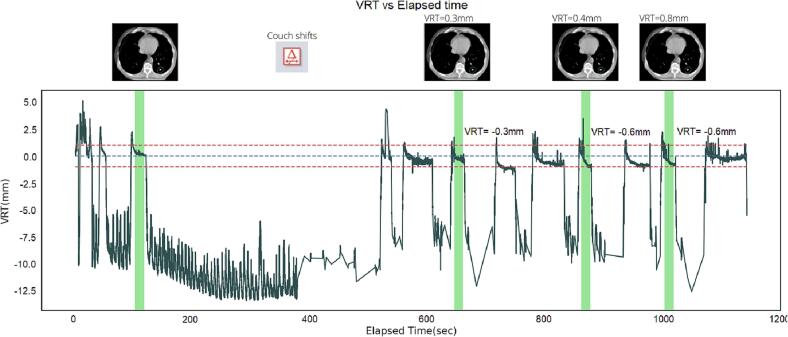
Respiratory motion information in the VRT direction in the function of time of one session. Green bars show the kV-CBCT apneas. (For interpretation of the references to colour in this figure legend, the reader is referred to the web version of this article.)

AlignRT InBore™’s automatically generated treatment reports which contains information on the surface position in all six DoF as a function of time, i.e. RTD reports, were retrospectively analysed using a Python [24] script with Pandas and NumPy libraries. Treatment timeline of each fraction extracted from Aria™ oncology information system (Varian Medical Systems, Palo Alto, USA) were matched with the elapsed time of the RTD reports to identify the BHs that correspond to control kV-CBCTs (cf. Fig. 1). For every control kV-CBCT BH, the median RTD value in each of the six DoF was computed, because RTD data is not normally distributed. Surface motion amplitude was similarly assessed from the difference between the maximal and minimal values of the median RTD position across the three control kV-CBCTs and compared to tumor motion amplitude. Reproducibility was also analysed considering patient gender to study the impact of topography on SGRT accuracy.

### 2.6 Statistical analysis

Dissimilarity in motion amplitude between upper, middle and lower-lobe tumors were assessed using independent samples *t*-test.

A linear mixed model was considered to investigate correlations between the amplitude of surface motion computed from RTD values with the amplitude of tumor motion computed from CBCT shifts. The RTD values were considered as a fixed factor and the variable patient as a within-participant random factor to find the correlation regardless of the patient and the variability across fractions. Linear regression coefficients and their confidence intervals were computed using pymer4 [25]. The agreement between the RTD values from AlignRT InBore™ and the CBCT shifts was quantified using the Bland and Altman Method [26]. The difference between the RTD values from AlignRT InBore™ and the CBCT shifts were plotted as a function of the mean RTD and CBCT value, considering each DoF. The mean of both measures and the 95% confidence interval limits were represented in the same plot.

Significant differences in PTV volumes between FB and DIBH were studied using *t*-test.

## 3 Results

### 3.1 Reproducibility of target position

For all patients, the median amplitude of tumor movement in DIBH ranged between 0 mm and 5 mm in the three translational directions, and between 0° to 3.3° in the three rotations. (as shown in Supplementary Material C). Considering target localisation and based on the analysis of 175 kv-CBCT, there was no difference in amplitude between upper, middle, and lower-lobe tumors (p > 0.05).

### 3.2 Reproducibility of surface position

Supplementary Material D documents the median amplitude (Q1 – Q3) of SGRT RTD values for each patient. The median surface position in DIBH remained within 0.9 (range: 0 – 4.3 mm) in the vertical direction, 1.3 (range: 0 – 7.1 mm) in the longitudinal direction, and 1 (range: 0 – 4.8 mm) in the lateral direction. The three rotations remained between 0° and 3.3°.

### 3.3 Correlation between surface and tumor position

**Fig. 2:**
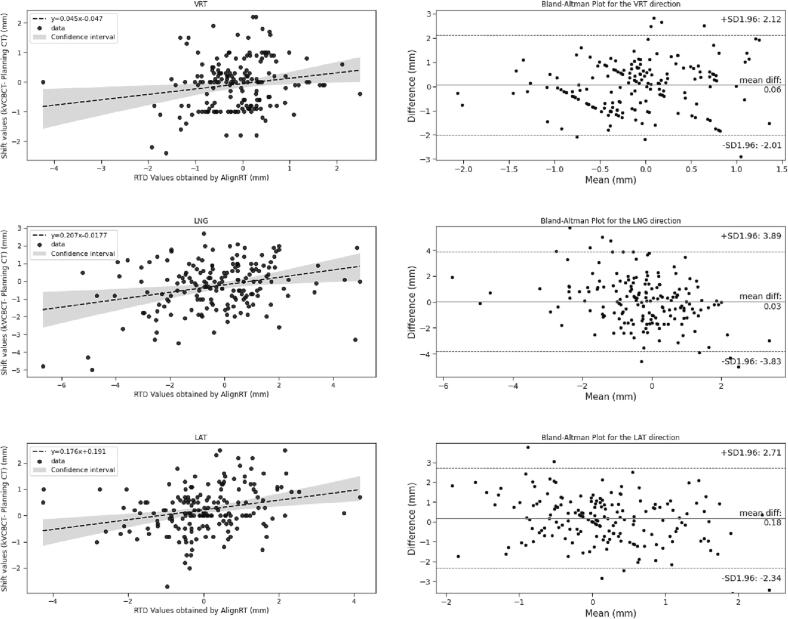
Left column shows the scatterplots with regression lines calculated using the Linear Mixed Model. The top and bottom dashed lines represent the 95% limits of agreement. The middle-dashed line is the mean of the difference between the RTD values and CBCT shifts. Right columns show the Bland and Altman Plots for the RTD values and CBCT shifts.

RTD values correlated with CBCT shifts in LNG (p < 0.001), LAT (p = 0.001) and ROLL (p = 0.05) directions, with a 95% confidence interval CI = [95% − 25%] of [0.3–0.1] mm, [0.3–0.1]mm and [0.4 – −0.01] mm, respectively. No relationship was obtained for the VRT, YAW and PITCH directions (cf. Fig. 2 and Supplementary Material D). Bland and Altman plots proved the mean difference between RTD values and CBCT shifts to remain < 0.03 mm [95% CI: −2, 2 mm] in the VRT direction and < 0.4 mm [95% CI: −4, 4 mm/°] in the other five DoF (cf. Fig. 2 and Supplementary Material E).

### 3.4 Benefits of using DIBH in lung SBRT dose delivery

The median ITV and GTV volumes were 11.1 cm^3^ (Q1-Q3: 6.8 – 18.9 cm^3^) and 5.1 cm^3^ (Q1-Q3: 3.9 – 12.9 cm^3^) respectively, showing an average ratio (GTV/ITV) of 56.4% (range: 40% - 77%). Consequently, the PTV of all targets decreased by 68% on average, with an median PTV value of 33.9 cm^3:^ (Q1-Q3: 22.8 – 54.8 cm^3^) in FB and 21.7 cm^3^(Q1-Q3:17.5 – 45.7cm^3^) in DIBH. PTV reduction consequently yielded considerable minimization of lung doses where the ratio between DIBH and FB mean lung dose and (D1%) maximal dose were on average 65.2% (range: 40.9% - 98.7%) and 73.6% (range: 38.3% - 95.4%), respectively. Lung volumes increased on average by 60.9% (sd = 12.6%) thanks to the DIBH procedure. On average, 8.7% (sd = 4.3%) of healthy lungs received 12.5 Gy if the treatment plan was done in FB. In contrast, 4.8% (sd = 2.4%) of healthy lungs received 12.5 Gy for DIBH plans overall, observing an average reduction of 59% (sd = 28%). A 58% (sd = 28%) decrease was also found for relative lung volumes receiving 13.5 Gy as a maximal dose.

## 4 Discussion

This work studied the average displacement of tumor and surface during repetitive DIBH manoeuvres to confirm the correlation of surface and internal anatomy and the robustness of using the latter as a good surrogate of the former. It also investigated the dose/volume benefits of SBRT lung treatments performed with the aDIBH technique using AlignRT InBore surface guidance system.

The analysis of 175 control kV-CBCT images showed that internal movement was limited thanks to DIBH, (<2.7 mm and 2°) with the tumor always remaining inside the PTV (5 mm isotropic) thanks to tight SGRT thresholds not only in the VRT direction (±1 mm) but also in the other five DoF (±2 mm/2°). The ability to acquire control CBCTs in a single DIBH represents a key benefit of combining SGRT and the closed-bore gantry linac for lung SBRT as opposed to the need for multiple BHs per CBCT acquisition (up to 3 BHs) on traditional C-arm linacs [11], [16].

Additionally, SGRT reports analysis showed that 90.7% of the RTD values remained in the established threshold window: ± 1 mm in the VRT, ± 2 mm/° in the other five DoF proving reproducible and stable voluntary aDIBH. However, the work highlights the importance of patient monitoring in six DoF since longitudinal tumor motion greater than 3.5 mm (couch shift of kV-CBCT) was observed in the third quartile (Q3) for patients 12 and 13. Previous literature also showed improved intra-fractional target position accuracy using six DoF surface tracking compared to one DoF RPM monitoring for Left Breast Irradiation [29]. For lung SBRT in DIBH, some papers have shown significant deviations in the other DoF post-CBCT/X-ray imaging [30].

It is important to mention that there were no significant differences (p greater than 0.05) in intrafraction reproducibility between male and female patients, despite the fact that male patients had lower topography in the treatment ROI. This finding contradicts previous research where SGRT was considered unsuitable for intrafraction target monitoring for male patients using a single camera ceiling-mounted SGRT system on C-arm linacs with sixDoF SGRT thresholds set to 4 mm [31]. Significant differences (p greater than 0.05) in amplitude of tumor movement were not found when considering target localization. This contrasts with previous studies, which showed that lower-lobe tumors moved more than upper-lobe tumors during free breathing [32] and deep inspiration breath-hold [7]. It should be considered that the smaller number of upper-lobe tumors (3 upper-lobe tumors vs 8 lower-lobe tumors) may introduce bias in the interpretation of our results.

The RTD data and CBCT shifts correlation was only observed for LNG, LAT and ROLL directions. This was expected since more than 90% of CBCT and RTD values in VRT, YAW and PITCH directions were in an interval [-1, 1] mm with almost all VRT values near zero thanks to the tight threshold window and patients’ visual coaching in DIBH in this direction. However, the Bland and Altman plots showed that the mean difference between RTD values and CBCT shifts remained < 5 mm, indicating that the tumor remained within the PTV whenever the surface position was within SGRT tolerance. These findings were agreed with previous literatures [11], [33] As such, it is safe to conclude that the ring-mounted SGRT system with tight thresholds is sufficient to monitor intrafraction tumor motion without the need for additional control kV-CBCT thereby improving patients’ radiation protection and saving treatment time.

It is worth noting that by combining a closed-bore gantry linac with a dedicated ring-mounted SGRT system, fast lung SBRT treatments in DIBH (22 min ± 4 min) can be achieved, even with the addition of three control kV-CBCT images. This is in contrast to typical respiratory gating lung SBRT treatments on C-arm linacs without systematic control CBCTs prior to each treatment arc/delivery, which can take 20–60 min [7], [8], [27].

Based on Table 1, it can be concluded that the optical performance of the SGRT system remains robust for tumors located up to 20 cm underneath the surface (SSD ranging from 80.1 to 91.7 cm). This result is comparable to the performance of the ceiling-mounted SGRT cameras [34], [35].

All patients experienced a considerable reduction in GTV (p = 0.008) and PTV volumes (p = 0.007), and doses to lung thanks to the DIBH technique (p = 0.006). Patients 1, 2, 4, 6 and 13 had a reduction of PTV volumes of more than 50%, while only two patients exhibited a reduction < 20%. Consequently, mean and maximal (12.5 Gy and 13.5 Gy) lung dose reduction were 65.2% and 55% respectively. These results are in agreement with previously reported data on lung and liver SBRT treatments using standard C-arm linacs, further highlighting the benefits of the DIBH technique [7], [8], [9], [27], [28].

As a single-center retrospective study, the present work presents several limitations owing to the limited number of patients. Additionally, the elevated number of DIBHs necessary to acquire the control CBCTs and treatment delivery (up to 15) limit the eligibility and require optimised treatment planning, with reduced number of MUs per arc and limiting the number of the control kV-CBCT. MUs were not optimized during this study but were rather collected and retrospectively analyzed to guide future planning. Using this analysis, we made efforts to significantly decrease the number of MUs for patients 1, 9, and 12 while upholding plan quality, including target volume coverage and organ-at-risk sparing. This was done with the aim of enhancing treatment efficiency and patient comfort. The work could not study reproducibility of lung-filling and intake volume since the kV-CBCT does not always allow for obtaining a complete lung image. Multi-variate analysis of tumor and surface motion as a function of target localisation, patient age and gender, chest elevation, etc. could not be performed due to the small sample. Further optimization of the ROI shape, size and location could also be considered.

Based on our experience, the ring-mounted SGRT system with tight thresholds in all 6 DoF allows a reproducible DIBH and precise tumor positioning during lung SBRT treatments on bore-based linacs.

## Declaration of Competing Interest

The authors declare that they have no known competing financial interests or personal relationships that could have appeared to influence the work reported in this paper.
