# Supplementary material for: Reproducibility of surface-based deep inspiration breath-hold technique for lung stereotactic body radiotherapy on a closed-bore gantry linac
Source: Phys Imaging Radiat Oncol. 2023 May 13;26:100448. doi: 10.1016/j.phro.2023.100448 (PMC10213090; doi:10.1016/j.phro.2023.100448)
Supplement: Supplementary Data 1 [file mmc1.docx]

Supplementary Material A


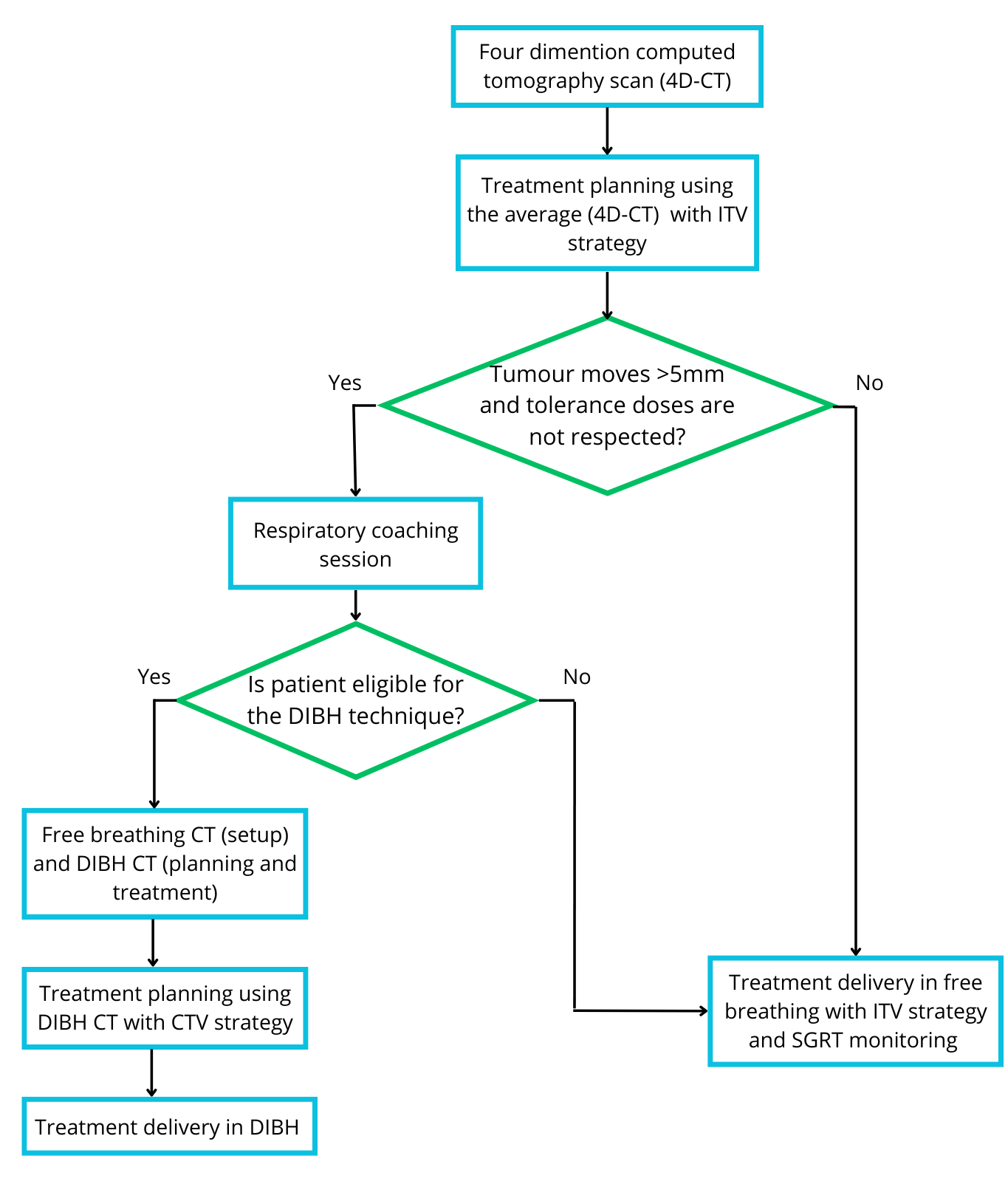


*SBRT Workflow for patient selection until treatment delivery. Abbreviations: DIBH: deep inspiration breath-hold. CT: Computed Tomography. ITV: Internal Target Volume. CTV: Clinical Target Volume.*

Supplementary Material B


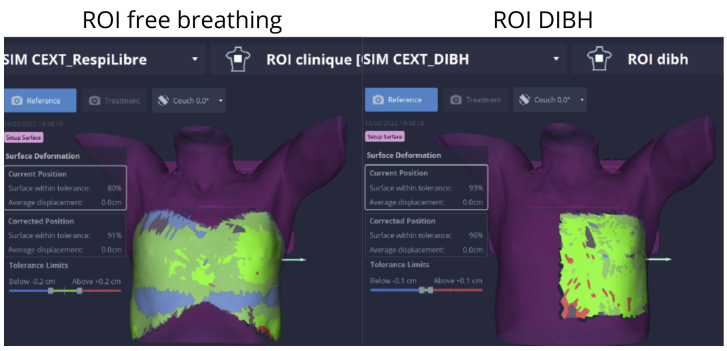


*Screenshot of AlignRT™ showing examples of two individual regions of interest (ROI) for setup in free-breathing (left) and treatment in DIBH (right).*

Supplementary Material C

| Patient No | VRT (mm) | LNG (mm) | LAT (mm) | ROLL (°) | YAW (°) | PITCH (°) |
| --- | --- | --- | --- | --- | --- | --- |
| 1 | 1.5 (0.9 - 1.7) | 0.6 (0.5 - 1.3) | 1.5 (1.1 - 2) | 1 (0.1- 2.2) | 0.5 (0.4 - 1) | 1.5 (0.9 - 1.5) |
| 2 | 1 (0.8 -1.1) | 1.7 (0.7 - 2.2) | 1.1 (0.6 - 1.7) | 0.8 (0.5 -1.3) | 1.2 (0.6 -1.7) | 1.1 (0.6 -1.3) |
| 3 | 1 (0.9 -1.2) | 1 (0.9 -1.7) | 1 (0.9 -1.1) | 1.5 (1.4 -1.7) | 1.1 (1.1 - 1.3) | 1.4 (1.1 - 1.5) |
| 4 | 0.3 (0.1 - 0.8) | 1 (1 - 1) | 0.2 (0.1 - 0.5) | 0.4 (0.3 - 0.5) | 0.6 (0.2 - 0.6) | 0.2 (0.1 - 0.5) |
| 5 | 0.2 (0 - 0.3) | 0.5 (0.3 - 0.5) | 0.5 (0.3 - 0.5) | 0.8 (0.1 - 0.8) | 0.4 (0.3 - 0.4) | 0.4 (0.1 - 0.5) |
| 6 | 0.7 (0.3 - 0.9) | 0.9 (0.6 - 1.3) | 0.5 (0 - 1.1) | 0.6 (0.1 - 1.1) | 0.9 (0.8 - 1.1) | 0.9 (0.7 -1.3) |
| 7 | 0.4 (0.1 - 0.7) | 1.2 (0.9 -1.4) | 1.3 (0.7 -1.8) | 0.4 (0.3 - 0.5) | 0.9 (0.6 -0.9) | 0.7 (0.5 -0.8) |
| 8 | 0.7 (0.3 - 0.9) | 1.2 (0.9 - 1.3) | 1 (0.9 - 1.1) | 0.6 (0.4 - 0.7) | 1 (0.3 - 1.4) | 0.9 (0.7 - 1.1) |
| 9 | 0.7 (0.5 - 1) | 1.2 (0.3 - 1.7) | 1 (0.6 - 1.4) | 0.6 (0.4 - 0.8) | 2 (1.8 - 2.1) | 1.3 (0.9 - 2.4) |
| 10 | 0.8 (0.6 - 1) | 1.4 (1.3 - 1.9) | 0.7 (0.6 - 1.3) | 0.4 (0.4 - 0.5) | 1.8 (0.8 - 1.8) | 1.3 (1 - 1.4) |
| 11 | 1.9 (1.6 -2.2) | 2.7 (2.7 -3.6) | 0.9 (0.8 -1.1) | 1.8 (1.2 - 1.9) | 0.9 (0.7 -1.1) | 1.4 (0.7 - 1.5) |
| 12 | 1.4 (1.3 - 1.7) | 2.7 (2.6 - 2.9) | 1.1 (1 - 2.2) | 1 (0.8 - 1.7) | 1.1 (1 - 1.2) | 2 (1.4 - 3) |
| 13 | 1.3 (1 - 1.6) | 2.6 (1.2 - 4.5) | 1.4 (1.4 - 1.5) | 1.3 (0.9 - 1.7) | 1.8 (1.4 - 1.8) | 1.4 (1 - 1.8) |

*Median (Q1-Q3) amplitude values of tumortumour motion in DIBH based on control CBCTs. Abbreviations: VRT: vertical (anterior-posterior), LNG: longitudinal (superior-inferior), LAT: lateral (left-right) directions.*

Supplementary Material D

| Patient No | VRT (mm) | LNG (mm) | LAT (mm) | ROLL (°) | YAW (°) | PITCH (°) |
| --- | --- | --- | --- | --- | --- | --- |
| 1 | 0.2 (0.1 - 0.3) | 2.5 (0.6 - 3.1) | 1 (0.11 - 1.5) | 1.1 (0.1 - 2.7) | 0.2 (0.1 - 0.8) | 0.3 (0.2 - 0.5) |
| 2 | 0.6 (0.2 - 0.8) | 3 (0.1 - 3.7) | 1 (0.1 - 3.2) | 0.5 (0.1 - 0.7) | 0.5 (0.2 - 1.1) | 1.7 (0.4 - 1.9) |
| 3 | 0.4 (0.1 - 0.6) | 1.4 (1.3 - 2) | 0.7 (0.4 - 0.7) | 0.2 (0.2 - 0.4) | 0.6 (0.3 - 0.9) | 0.3 (0.2 - 0.4) |
| 4 | 0.6 (0.3 - 0.9) | 1.1 (0.6 - 2) | 1.3 (1.2 - 2.5) | 0.5 (0.2 - 0.6) | 0.5 (0.4 - 0.8) | 0.2 (0.2 - 0.5) |
| 5 | 0.4 (0.3 - 0.5) | 0.3 (0.2 - 0.4) | 0.5 (0.2 - 0.9) | 0 (0 - 0.1) | 0.2 (0.1 - 0.5) | 0.3 (0.1 - 0.3) |
| 6 | 1.1 (0.3 - 2.3) | 0.4 (0.1 - 1.6) | 0.8 (0.3 - 1.3) | 0.6 (0.3 - 0.7) | 0.7 (0.3 - 1) | 0.5 (0.1 - 1) |
| 7 | 0.4 (0.1 - 1.2) | 1.6 (0.4 - 2.6) | 1.4 (0.1 - 2.9) | 0.2 (0.1 - 0.4) | 0.4 (0.1 - 0.8) | 0.2 (0.1 - 0.7) |
| 8 | 0.7 (0.2 - 1.3) | 1.1 (0.5 - 1.9) | 1.9 (0.8 - 2.2) | 0.3 (0.2 - 0.3) | 0.6 (0.4 - 0.8) | 0.2 (0 - 0.4) |
| 9 | 0.3 (0 - 0.5) | 1.4 (0.2 - 2.8) | 0.5 (0 - 1) | 0.2 (0.1 - 0.3) | 0.1 (0.1 - 0.3) | 0.8 (0.4 - 1) |
| 10 | 0.1 (0 - 0.4) | 1.3 (1 - 1.5) | 0.1 (0.1 - 0.4) | 0.1 (0.1 - 0.2) | 0.1 (0 - 0.3) | 0.2 (0.2 - 0.3) |
| 11 | 0.8 (0.5 -1) | 1.6 (1.5 - 1.8) | 0.6 (0.4 - 0.9) | 0.2 (0.2 - 0.3) | 0.5 (0.1 - 0.6) | 0.4 (0.1 -0.5) |
| 12 | 1.1 ( 0.8 - 1.8) | 2.6 (1.9 - 3.4) | 2.1 (2.6 - 1.5) | 0.7 (0.7 - 0.8) | 0.3 (0.2 - 0.8) | 0.7 (0.1 - 0.9) |
| 13 | 1.3 (1 - 1.5) | 2.3 (1.2 - 5.8) | 1.1 (1 - 1.3) | 0.6 (0.6 - 0.7) | 0.6 (0.4 - 0.7) | 0.5 (0.3 - 0.6) |

*Median (Q1 – Q3) amplitude values of SGRT surface position in DIBH during the control CBCTs. Abbreviations: VRT: vertical (anterior-posterior), LNG: longitudinal (superior-inferior), LAT: lateral (left-right) directions.*

**Supplementary** Material  **E**


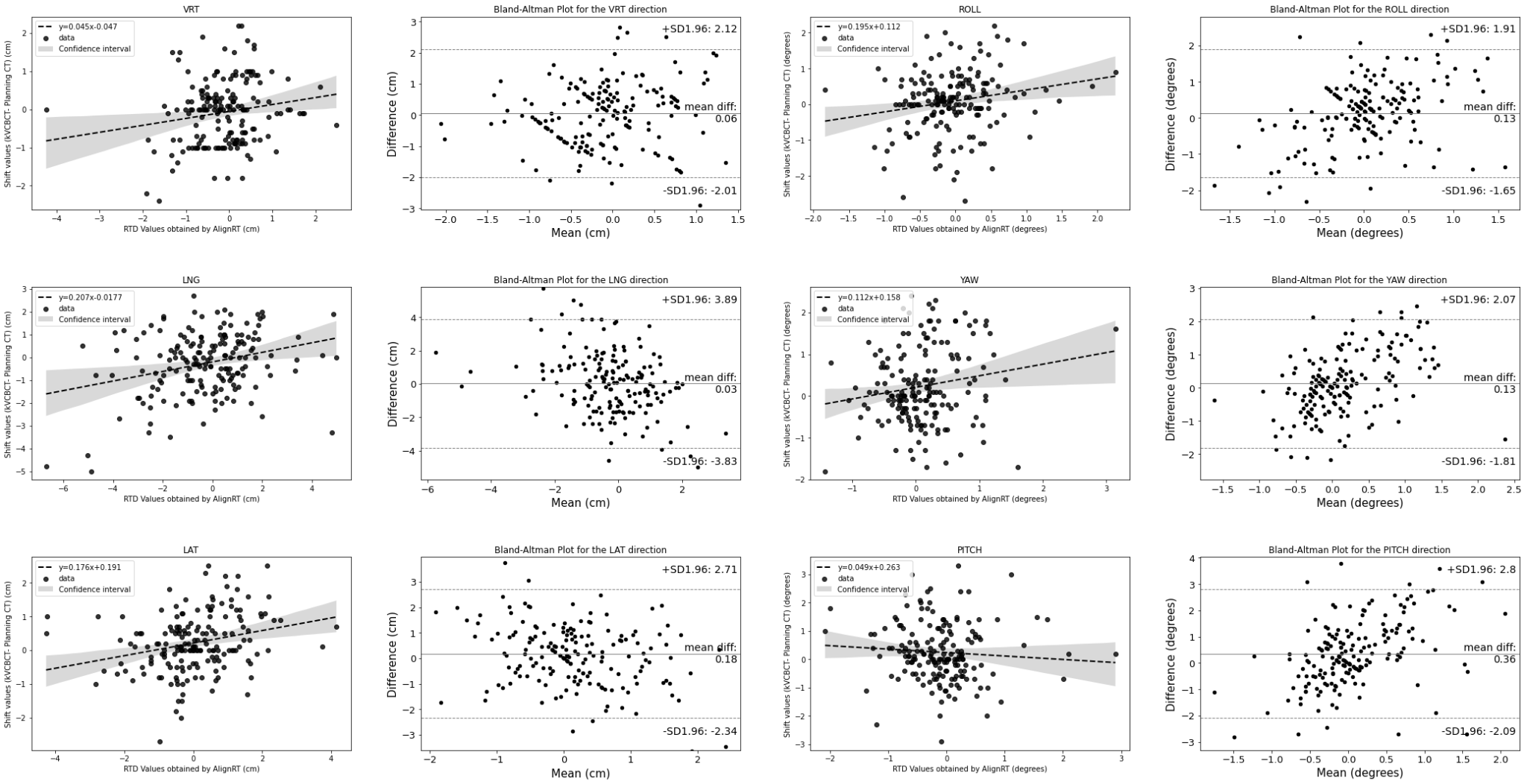


*Odd column shows the scatterplots with regression lines calculated using the Linear Mixed Model for the rotational directions. The top and bottom dashed lines represent the 95% limits of agreement. The middle-dashed line is the mean of the difference between the RTD values and CBCT shifts. Even columns exhibit the Bland and Altman Plots for the RTD values and CBCT shifts or the rotational directions.*
